# Finances of the Texas State Medical Association

**Published:** 1890-08

**Authors:** F. E. Daniel

**Affiliations:** Secretary; Texas State Medical Association, Office of Secretary, Austin, Tex.


### EDITORIAL DEPARTMENT.

F. E. DANIEL, M. D., EDITOR AND PUBLISHER.

THIS JOURNAL, by unanimous vote of the members, was made the Official Organ of the Austin District Medical Society.

COLLABORATORS:

*Dr. R. M. Swearingen, Austin.*
*Prof. B. E. Hadra, M. D., Galveston.*
*Prof. Geo. Cupples, M. D., San Antonio.*
*Dr. T. C. Osborn, Cleburne, Texas.*
*Dr E. J. Doering, Chicago.*
*Dr. E. J. Beall, Fort Worth.*
*Dr Odo Betz, Germany.*
*Prof. W. B. Rogers, M. D., Memphis.*
*Dr. J. W. McLaughlin, Austin.*
*Dr. T. J. Tyner, Austin.*
*Prof. J. F. Y. Paine, M. D., Galveston.*
*Dr. R. H. L. Bibb, Mexico.*
*Dr. T. J. Bennett. Austin.*
*Dr. Bat Smith, Wharton.*
*Dr. E. Meierhof, New York.*
*L. H. Luce, M. D., West Tisbury, Mass.*

### FINANCES OF THE TEXAS STATE MEDICAL ASSOCIATION.

The by-laws of the Texas State Medical Association says "the Secretary shall keep the accounts with the members, collect all moneys due the Association, and turn it over to the Treasurer."

This requirement, so far as we are aware, has never been fulfilled. From earliest times—some nineteen years, we believe—that being the length of time that our efficient Treasurer has served the Association, the Treasurer has kept the accounts and has received all moneys due the Association. The exception has been, heretofore, that a remittance has been made to the Secretary.

When the present incumbent of the office of Secretary came into office he found the status as above—the Treasurer keeping the accounts—and he, of course, fell into the custom, reporting such funds as came into his hands, and accounting for what was expended in his office. Hence, members believing that the Secretary keeps the accounts, as required by the by-laws, hold him responsible for all errors in said accounts, and very unjustly. As to how and why the system, as obtains now, ever begun, we do not know. The by-law, requiring the Secretary to keep this account, should be repealed,—it has never been in force and cannot be enforced;—for any member who has ever attended a meeting of the Association, will readily understand how impossible it would be for the Secretary to do what is done by the Treasurer on these occasions, especially in addition to his own arduous and absorbing work during the four days' sessions. To do so, were it possible, would practically abolish the office of Treasurer.

At the last meeting a resolution was adopted which prohibits the Secretary from sending the Transactions to those members who have not paid their dues for the current year, 1890–1. There is also a standing law which says "members in arrears for dues three years or over *shall stand suspended;* and shall be reinstated only upon paying up all arrears; in default of which they shall be dropped from the roll." This was amended by the adoption, at Dallas, in 1886, of a resolution which says "any member whose name has been dropped from the roll for non-payment of dues, may be reinstated upon the payment of $10—the amount required of new members on admission." Of course, that means, $10 in lieu of all back dues; it puts said members on a footing with new members—except he is (re-) admitted without application and reference to the Judicial Council.

After the meeting at Dallas, in 1886, sixty members were dropped from the roll for non-payment of dues. For the next four years the Secretary *did not* carry out this by-law—and none have been dropped since 1886. But after the meeting at Fort Worth it was ascertained that there were a great many who had paid nothing, absolutely, in three and four years—and that less than half of the members were paying the expenses of the Association, and that the roll was encumbered by a very large number of, practically, dead beats; it was determined to induce them to pay up, or drop them; hence, the Secretary wrote to the Treasurer for a list both of the three and four years delinquents—suspended members,—with a view of erasing their names from the roll—and a list of those in arrears for less than three years. Instead of furnishing the Secretary with such lists, the Treasurer sent up his books by express, and the Secretary made the lists for hmself. In consequence of a misunderstanding of the entries of credits made by the Treasurer, the Secretary did some members injustice by sending them bills, in some cases, for $5 more than they owed, and bills to some others who had paid in full and held the Treasurer's receipt. Some took offense,—some,—some of the smaller and more narrow-minded,—wrote back insulting letters, but the majority simply informed the Secretary that they had Dr. Larendon's receipt and there must be some mistake.

The trouble arose in this way: When a member joins he pays an initiation fee of $5, and a year's dues, $5, in advance. At each meeting, if he pays, he keeps one year in advance, and it should so appear on the books, but it does not. For instance, a member, who, at the San Antonio meeting (1889) owed for the *past* year, 1888, and '89, and paid that, and the year ahead—$10, —the entry made by the Treasurer was, "1889—paid $10," whereas it should appear that he had paid up to and including the year ending April, 1890. Well, the Secretary, seeing the above entry, naturally supposed that this member had paid his dues only up to the year ending April, 1889! Hence, the member was notified that he owed dues from 1889 to 1891—two years. The obliging Treasurer very cheerfully acknowledges that it is his fault, and says next time he will send a list.

This explanation is made for the benefit of those, who, having paid, were subjected to the mortification of a *dun*.

The Secretary issued the following circulars:

TEXAS STATE MEDICAL ASSOCIATION,
OFFICE OF SECRETARY, AUSTIN, TEX., July 10, 1890.

DEAR DOCTOR:—Under the By-laws of the Association you stand suspended for non-payment of dues for three years. Unless remittance be made by August 15th you will be dropped from the roll and your name will not appear in the forthcoming Transactions; nor will you be entitled to receive a copy. By resolution passed at Dallas, in 1886, you can be re-instated upon the payment of TEN DOLLARS—the same amount that is paid by new members upon admittance. Remit to Dr. J. Larendon, Treasurer, Houston, or to the undersigned.

Very respectfully yours,
F. E. DANIEL, M. D., Secretary.

This circular was sent to one hundred and ten members. Up to date of going to press about ten have been re-instated.

TEXAS STATE MEDICAL ASSOCIATION,
OFFICE OF SECRETARY, AUSTIN, TEX., July 10, 1890.

DEAR DOCTOR:—You being in arrears to the Association for dues since————, by resolutions adopted at Fort Worth in April last, I am forbidden to mail you the Transactions for this year, now in press. If you wish to get the work and to "tote level" with the other members, please remit before the 15th of August, either to Dr. J. Larendon or to me.

The amount due to April, 1891 (one year required in advance) is $————.

Very respectfully yours

F. E. DANIEL, M. D., Secretary.

No. 2 was sent to two hundred and fifty-six members; but, in many instances, it was an error. Suffice it to say, they are paying up, and to date some four hundred dollars of the delinquent dues has been paid; nearly or quite enough to pay for the Transactions.

In this connection we will state for the information of members, that the Transactions will not be ready before the latter part of September. The printers take the contract at the very low price that they do, with the understanding that the work can be done in the more leisure time,—other work having preference.

The JOURNAL claims some credit for the present state of the treasury; never before has it had so much money on hand after paying all claims, and it is because the JOURNAL insisted that the members be made to pay their dues, and suggested that badges and Transactions be given only to those who do pay; and it will get better still. After all the deadheads are eliminated, and a new element is added to the paying element already existing, the payment of dues each year, will be a matter of course. —We may say that this year—after paying all bills, including Transactions —there will be between $700 to $1,000 in the treasury. What shall we do with it?

---

A solution of chloral hydrate, five grains to the ounce of water, will clear the hair of dandruff and prevents its falling out from this cause.—*American Lancet.*

